# Ensembl 2025

**DOI:** 10.1093/nar/gkae1071

**Published:** 2024-12-04

**Authors:** Sarah C Dyer, Olanrewaju Austine-Orimoloye, Andrey G Azov, Matthieu Barba, If Barnes, Vianey Paola Barrera-Enriquez, Arne Becker, Ruth Bennett, Martin Beracochea, Andrew Berry, Jyothish Bhai, Simarpreet Kaur Bhurji, Sanjay Boddu, Paulo R Branco Lins, Lucy Brooks, Shashank Budhanuru Ramaraju, Lahcen I Campbell, Manuel Carbajo Martinez, Mehrnaz Charkhchi, Lucas A Cortes, Claire Davidson, Sukanya Denni, Kamalkumar Dodiya, Sarah Donaldson, Bilal El Houdaigui, Tamara El Naboulsi, Oluwadamilare Falola, Reham Fatima, Thiago Genez, Jose Gonzalez Martinez, Tatiana Gurbich, Matthew Hardy, Zoe Hollis, Toby Hunt, Mike Kay, Vinay Kaykala, Diana Lemos, Disha Lodha, Nourhen Mathlouthi, Gabriela Alejandra Merino, Ryan Merritt, Louisse Paola Mirabueno, Aleena Mushtaq, Syed Nakib Hossain, José G Pérez-Silva, Malcolm Perry, Ivana Piližota, Daniel Poppleton, Irina Prosovetskaia, Shriya Raj, Ahamed Imran Abdul Salam, Shradha Saraf, Nuno Saraiva-Agostinho, Swati Sinha, Botond Sipos, Vasily Sitnik, Emily Steed, Marie-Marthe Suner, Likhitha Surapaneni, Kyösti Sutinen, Francesca Floriana Tricomi, Ian Tsang, David Urbina-Gómez, Andres Veidenberg, Thomas A Walsh, Natalie L Willhoft, Jamie Allen, Jorge Alvarez-Jarreta, Marc Chakiachvili, Jitender Cheema, Jorge Batista da Rocha, Nishadi H De Silva, Stefano Giorgetti, Leanne Haggerty, Garth R Ilsley, Jon Keatley, Jane E Loveland, Benjamin Moore, Jonathan M Mudge, Guy Naamati, John Tate, Stephen J Trevanion, Andrea Winterbottom, Bethany Flint, Adam Frankish, Sarah E Hunt, Robert D Finn, Mallory A Freeberg, Peter W Harrison, Fergal J Martin, Andrew D Yates

**Affiliations:** European Molecular Biology Laboratory, European Bioinformatics Institute (EMBL-EBI), Wellcome Genome Campus, Hinxton, Cambridge CB10 1SD, UK; European Molecular Biology Laboratory, European Bioinformatics Institute (EMBL-EBI), Wellcome Genome Campus, Hinxton, Cambridge CB10 1SD, UK; European Molecular Biology Laboratory, European Bioinformatics Institute (EMBL-EBI), Wellcome Genome Campus, Hinxton, Cambridge CB10 1SD, UK; European Molecular Biology Laboratory, European Bioinformatics Institute (EMBL-EBI), Wellcome Genome Campus, Hinxton, Cambridge CB10 1SD, UK; European Molecular Biology Laboratory, European Bioinformatics Institute (EMBL-EBI), Wellcome Genome Campus, Hinxton, Cambridge CB10 1SD, UK; European Molecular Biology Laboratory, European Bioinformatics Institute (EMBL-EBI), Wellcome Genome Campus, Hinxton, Cambridge CB10 1SD, UK; European Molecular Biology Laboratory, European Bioinformatics Institute (EMBL-EBI), Wellcome Genome Campus, Hinxton, Cambridge CB10 1SD, UK; European Molecular Biology Laboratory, European Bioinformatics Institute (EMBL-EBI), Wellcome Genome Campus, Hinxton, Cambridge CB10 1SD, UK; European Molecular Biology Laboratory, European Bioinformatics Institute (EMBL-EBI), Wellcome Genome Campus, Hinxton, Cambridge CB10 1SD, UK; European Molecular Biology Laboratory, European Bioinformatics Institute (EMBL-EBI), Wellcome Genome Campus, Hinxton, Cambridge CB10 1SD, UK; European Molecular Biology Laboratory, European Bioinformatics Institute (EMBL-EBI), Wellcome Genome Campus, Hinxton, Cambridge CB10 1SD, UK; European Molecular Biology Laboratory, European Bioinformatics Institute (EMBL-EBI), Wellcome Genome Campus, Hinxton, Cambridge CB10 1SD, UK; European Molecular Biology Laboratory, European Bioinformatics Institute (EMBL-EBI), Wellcome Genome Campus, Hinxton, Cambridge CB10 1SD, UK; European Molecular Biology Laboratory, European Bioinformatics Institute (EMBL-EBI), Wellcome Genome Campus, Hinxton, Cambridge CB10 1SD, UK; European Molecular Biology Laboratory, European Bioinformatics Institute (EMBL-EBI), Wellcome Genome Campus, Hinxton, Cambridge CB10 1SD, UK; European Molecular Biology Laboratory, European Bioinformatics Institute (EMBL-EBI), Wellcome Genome Campus, Hinxton, Cambridge CB10 1SD, UK; European Molecular Biology Laboratory, European Bioinformatics Institute (EMBL-EBI), Wellcome Genome Campus, Hinxton, Cambridge CB10 1SD, UK; European Molecular Biology Laboratory, European Bioinformatics Institute (EMBL-EBI), Wellcome Genome Campus, Hinxton, Cambridge CB10 1SD, UK; European Molecular Biology Laboratory, European Bioinformatics Institute (EMBL-EBI), Wellcome Genome Campus, Hinxton, Cambridge CB10 1SD, UK; European Molecular Biology Laboratory, European Bioinformatics Institute (EMBL-EBI), Wellcome Genome Campus, Hinxton, Cambridge CB10 1SD, UK; European Molecular Biology Laboratory, European Bioinformatics Institute (EMBL-EBI), Wellcome Genome Campus, Hinxton, Cambridge CB10 1SD, UK; European Molecular Biology Laboratory, European Bioinformatics Institute (EMBL-EBI), Wellcome Genome Campus, Hinxton, Cambridge CB10 1SD, UK; Université de Rouen Normandie, UFR Sciences et Techniques, 3 Av. Pasteur, 76000 Rouen, France; European Molecular Biology Laboratory, European Bioinformatics Institute (EMBL-EBI), Wellcome Genome Campus, Hinxton, Cambridge CB10 1SD, UK; European Molecular Biology Laboratory, European Bioinformatics Institute (EMBL-EBI), Wellcome Genome Campus, Hinxton, Cambridge CB10 1SD, UK; European Molecular Biology Laboratory, European Bioinformatics Institute (EMBL-EBI), Wellcome Genome Campus, Hinxton, Cambridge CB10 1SD, UK; European Molecular Biology Laboratory, European Bioinformatics Institute (EMBL-EBI), Wellcome Genome Campus, Hinxton, Cambridge CB10 1SD, UK; European Molecular Biology Laboratory, European Bioinformatics Institute (EMBL-EBI), Wellcome Genome Campus, Hinxton, Cambridge CB10 1SD, UK; European Molecular Biology Laboratory, European Bioinformatics Institute (EMBL-EBI), Wellcome Genome Campus, Hinxton, Cambridge CB10 1SD, UK; European Molecular Biology Laboratory, European Bioinformatics Institute (EMBL-EBI), Wellcome Genome Campus, Hinxton, Cambridge CB10 1SD, UK; European Molecular Biology Laboratory, European Bioinformatics Institute (EMBL-EBI), Wellcome Genome Campus, Hinxton, Cambridge CB10 1SD, UK; European Molecular Biology Laboratory, European Bioinformatics Institute (EMBL-EBI), Wellcome Genome Campus, Hinxton, Cambridge CB10 1SD, UK; European Molecular Biology Laboratory, European Bioinformatics Institute (EMBL-EBI), Wellcome Genome Campus, Hinxton, Cambridge CB10 1SD, UK; European Molecular Biology Laboratory, European Bioinformatics Institute (EMBL-EBI), Wellcome Genome Campus, Hinxton, Cambridge CB10 1SD, UK; European Molecular Biology Laboratory, European Bioinformatics Institute (EMBL-EBI), Wellcome Genome Campus, Hinxton, Cambridge CB10 1SD, UK; European Molecular Biology Laboratory, European Bioinformatics Institute (EMBL-EBI), Wellcome Genome Campus, Hinxton, Cambridge CB10 1SD, UK; European Molecular Biology Laboratory, European Bioinformatics Institute (EMBL-EBI), Wellcome Genome Campus, Hinxton, Cambridge CB10 1SD, UK; European Molecular Biology Laboratory, European Bioinformatics Institute (EMBL-EBI), Wellcome Genome Campus, Hinxton, Cambridge CB10 1SD, UK; European Molecular Biology Laboratory, European Bioinformatics Institute (EMBL-EBI), Wellcome Genome Campus, Hinxton, Cambridge CB10 1SD, UK; European Molecular Biology Laboratory, European Bioinformatics Institute (EMBL-EBI), Wellcome Genome Campus, Hinxton, Cambridge CB10 1SD, UK; European Molecular Biology Laboratory, European Bioinformatics Institute (EMBL-EBI), Wellcome Genome Campus, Hinxton, Cambridge CB10 1SD, UK; European Molecular Biology Laboratory, European Bioinformatics Institute (EMBL-EBI), Wellcome Genome Campus, Hinxton, Cambridge CB10 1SD, UK; European Molecular Biology Laboratory, European Bioinformatics Institute (EMBL-EBI), Wellcome Genome Campus, Hinxton, Cambridge CB10 1SD, UK; European Molecular Biology Laboratory, European Bioinformatics Institute (EMBL-EBI), Wellcome Genome Campus, Hinxton, Cambridge CB10 1SD, UK; European Molecular Biology Laboratory, European Bioinformatics Institute (EMBL-EBI), Wellcome Genome Campus, Hinxton, Cambridge CB10 1SD, UK; European Molecular Biology Laboratory, European Bioinformatics Institute (EMBL-EBI), Wellcome Genome Campus, Hinxton, Cambridge CB10 1SD, UK; European Molecular Biology Laboratory, European Bioinformatics Institute (EMBL-EBI), Wellcome Genome Campus, Hinxton, Cambridge CB10 1SD, UK; European Molecular Biology Laboratory, European Bioinformatics Institute (EMBL-EBI), Wellcome Genome Campus, Hinxton, Cambridge CB10 1SD, UK; European Molecular Biology Laboratory, European Bioinformatics Institute (EMBL-EBI), Wellcome Genome Campus, Hinxton, Cambridge CB10 1SD, UK; European Molecular Biology Laboratory, European Bioinformatics Institute (EMBL-EBI), Wellcome Genome Campus, Hinxton, Cambridge CB10 1SD, UK; European Molecular Biology Laboratory, European Bioinformatics Institute (EMBL-EBI), Wellcome Genome Campus, Hinxton, Cambridge CB10 1SD, UK; European Molecular Biology Laboratory, European Bioinformatics Institute (EMBL-EBI), Wellcome Genome Campus, Hinxton, Cambridge CB10 1SD, UK; European Molecular Biology Laboratory, European Bioinformatics Institute (EMBL-EBI), Wellcome Genome Campus, Hinxton, Cambridge CB10 1SD, UK; European Molecular Biology Laboratory, European Bioinformatics Institute (EMBL-EBI), Wellcome Genome Campus, Hinxton, Cambridge CB10 1SD, UK; European Molecular Biology Laboratory, European Bioinformatics Institute (EMBL-EBI), Wellcome Genome Campus, Hinxton, Cambridge CB10 1SD, UK; European Molecular Biology Laboratory, European Bioinformatics Institute (EMBL-EBI), Wellcome Genome Campus, Hinxton, Cambridge CB10 1SD, UK; European Molecular Biology Laboratory, European Bioinformatics Institute (EMBL-EBI), Wellcome Genome Campus, Hinxton, Cambridge CB10 1SD, UK; European Molecular Biology Laboratory, European Bioinformatics Institute (EMBL-EBI), Wellcome Genome Campus, Hinxton, Cambridge CB10 1SD, UK; European Molecular Biology Laboratory, European Bioinformatics Institute (EMBL-EBI), Wellcome Genome Campus, Hinxton, Cambridge CB10 1SD, UK; European Molecular Biology Laboratory, European Bioinformatics Institute (EMBL-EBI), Wellcome Genome Campus, Hinxton, Cambridge CB10 1SD, UK; European Molecular Biology Laboratory, European Bioinformatics Institute (EMBL-EBI), Wellcome Genome Campus, Hinxton, Cambridge CB10 1SD, UK; European Molecular Biology Laboratory, European Bioinformatics Institute (EMBL-EBI), Wellcome Genome Campus, Hinxton, Cambridge CB10 1SD, UK; European Molecular Biology Laboratory, European Bioinformatics Institute (EMBL-EBI), Wellcome Genome Campus, Hinxton, Cambridge CB10 1SD, UK; NIAB, Lawrence Weaver Road, Cambridge CB3 0LE, UK; University of Nottingham, Department of Plant Science, Plant Sciences Building, Sutton Bonnington Campus, Nottingham LE12 5RD, UK; European Molecular Biology Laboratory, European Bioinformatics Institute (EMBL-EBI), Wellcome Genome Campus, Hinxton, Cambridge CB10 1SD, UK; European Molecular Biology Laboratory, European Bioinformatics Institute (EMBL-EBI), Wellcome Genome Campus, Hinxton, Cambridge CB10 1SD, UK; European Molecular Biology Laboratory, European Bioinformatics Institute (EMBL-EBI), Wellcome Genome Campus, Hinxton, Cambridge CB10 1SD, UK; European Molecular Biology Laboratory, European Bioinformatics Institute (EMBL-EBI), Wellcome Genome Campus, Hinxton, Cambridge CB10 1SD, UK; European Molecular Biology Laboratory, European Bioinformatics Institute (EMBL-EBI), Wellcome Genome Campus, Hinxton, Cambridge CB10 1SD, UK; European Molecular Biology Laboratory, European Bioinformatics Institute (EMBL-EBI), Wellcome Genome Campus, Hinxton, Cambridge CB10 1SD, UK; European Molecular Biology Laboratory, European Bioinformatics Institute (EMBL-EBI), Wellcome Genome Campus, Hinxton, Cambridge CB10 1SD, UK; European Molecular Biology Laboratory, European Bioinformatics Institute (EMBL-EBI), Wellcome Genome Campus, Hinxton, Cambridge CB10 1SD, UK; European Molecular Biology Laboratory, European Bioinformatics Institute (EMBL-EBI), Wellcome Genome Campus, Hinxton, Cambridge CB10 1SD, UK; European Molecular Biology Laboratory, European Bioinformatics Institute (EMBL-EBI), Wellcome Genome Campus, Hinxton, Cambridge CB10 1SD, UK; European Molecular Biology Laboratory, European Bioinformatics Institute (EMBL-EBI), Wellcome Genome Campus, Hinxton, Cambridge CB10 1SD, UK; European Molecular Biology Laboratory, European Bioinformatics Institute (EMBL-EBI), Wellcome Genome Campus, Hinxton, Cambridge CB10 1SD, UK; European Molecular Biology Laboratory, European Bioinformatics Institute (EMBL-EBI), Wellcome Genome Campus, Hinxton, Cambridge CB10 1SD, UK; European Molecular Biology Laboratory, European Bioinformatics Institute (EMBL-EBI), Wellcome Genome Campus, Hinxton, Cambridge CB10 1SD, UK; European Molecular Biology Laboratory, European Bioinformatics Institute (EMBL-EBI), Wellcome Genome Campus, Hinxton, Cambridge CB10 1SD, UK; European Molecular Biology Laboratory, European Bioinformatics Institute (EMBL-EBI), Wellcome Genome Campus, Hinxton, Cambridge CB10 1SD, UK; European Molecular Biology Laboratory, European Bioinformatics Institute (EMBL-EBI), Wellcome Genome Campus, Hinxton, Cambridge CB10 1SD, UK; European Molecular Biology Laboratory, European Bioinformatics Institute (EMBL-EBI), Wellcome Genome Campus, Hinxton, Cambridge CB10 1SD, UK; European Molecular Biology Laboratory, European Bioinformatics Institute (EMBL-EBI), Wellcome Genome Campus, Hinxton, Cambridge CB10 1SD, UK; European Molecular Biology Laboratory, European Bioinformatics Institute (EMBL-EBI), Wellcome Genome Campus, Hinxton, Cambridge CB10 1SD, UK; European Molecular Biology Laboratory, European Bioinformatics Institute (EMBL-EBI), Wellcome Genome Campus, Hinxton, Cambridge CB10 1SD, UK; European Molecular Biology Laboratory, European Bioinformatics Institute (EMBL-EBI), Wellcome Genome Campus, Hinxton, Cambridge CB10 1SD, UK; European Molecular Biology Laboratory, European Bioinformatics Institute (EMBL-EBI), Wellcome Genome Campus, Hinxton, Cambridge CB10 1SD, UK; European Molecular Biology Laboratory, European Bioinformatics Institute (EMBL-EBI), Wellcome Genome Campus, Hinxton, Cambridge CB10 1SD, UK; European Molecular Biology Laboratory, European Bioinformatics Institute (EMBL-EBI), Wellcome Genome Campus, Hinxton, Cambridge CB10 1SD, UK; European Molecular Biology Laboratory, European Bioinformatics Institute (EMBL-EBI), Wellcome Genome Campus, Hinxton, Cambridge CB10 1SD, UK; European Molecular Biology Laboratory, European Bioinformatics Institute (EMBL-EBI), Wellcome Genome Campus, Hinxton, Cambridge CB10 1SD, UK; European Molecular Biology Laboratory, European Bioinformatics Institute (EMBL-EBI), Wellcome Genome Campus, Hinxton, Cambridge CB10 1SD, UK; European Molecular Biology Laboratory, European Bioinformatics Institute (EMBL-EBI), Wellcome Genome Campus, Hinxton, Cambridge CB10 1SD, UK

## Abstract

Ensembl (www.ensembl.org) is an open platform integrating publicly available genomics data across the tree of life with a focus on eukaryotic species related to human health, agriculture and biodiversity. This year has seen a continued expansion in the number of species represented, with >4800 eukaryotic and >31 300 prokaryotic genomes available. The new Ensembl site, currently in beta, has continued to develop, currently holding >2700 eukaryotic genome assemblies. The new site provides genome, gene, transcript, homology and variation views, and will replace the current Rapid Release site; this represents a key step towards provision of a single integrated Ensembl site. Additional activities have included developing improved regulatory annotation for human, mouse and agricultural species, and expanding the Ensembl Variant Effect Predictor tool. To learn more about Ensembl, help and documentation are available along with an extensive training program that can be accessed via our training pages.

## Graphical Abstract

**Graphical Abstract:**
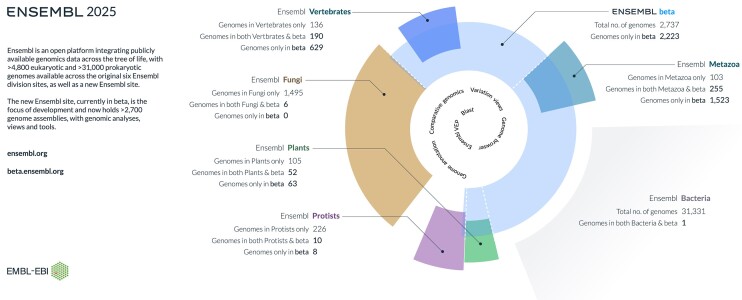

## Introduction

The Ensembl platform integrates and analyses publicly available genomics data from both eukaryotes and prokaryotes, underpinning a range of use cases from basic research to clinical and agricultural applications. The data included are focused on genomes, genomic annotations including gene models and functional predictions, genetic variants and links to relevant external resources. Gene prediction, functional annotation, regulatory region predictions and comparative analyses are performed in-house, using Ensembl’s open source pipelines. In addition, key community-generated annotations are also imported for a number of species. Data can be accessed via ensembl.org, using BioMart, programmatically with the REST API, from our public MySQL server and also our FTP site, where standard file formats and Ensembl MySQL databases are available.

Ensembl is currently comprised of seven component sites: ensembl.org for vertebrate genomes, including human and mouse reference assemblies; metazoa.ensembl.org for invertebrates; and plants.ensembl.org, fungi.ensembl.org, protists.ensembl.org and bacteria.ensembl.org for plant, fungi, protists and bacteria/archaea, respectively. Ensembl’s Rapid Release site, rapid.ensembl.org, was established in 2020 to provide a route to speedily deploy a lightweight version of Ensembl for genomes coming from global biodiversity and pangenome projects. The final data release for the Rapid Release site occurred in September 2024, with all data mirrored on beta.ensembl.org, the new home for ‘Rapid’ releases, or in the component sites. We will provide a reduced functionality archive of Rapid Release until mid-2025, when we expect all Rapid Release users to have transitioned to our new infrastructure.

## Diversity of genomes

**Figure 1.**
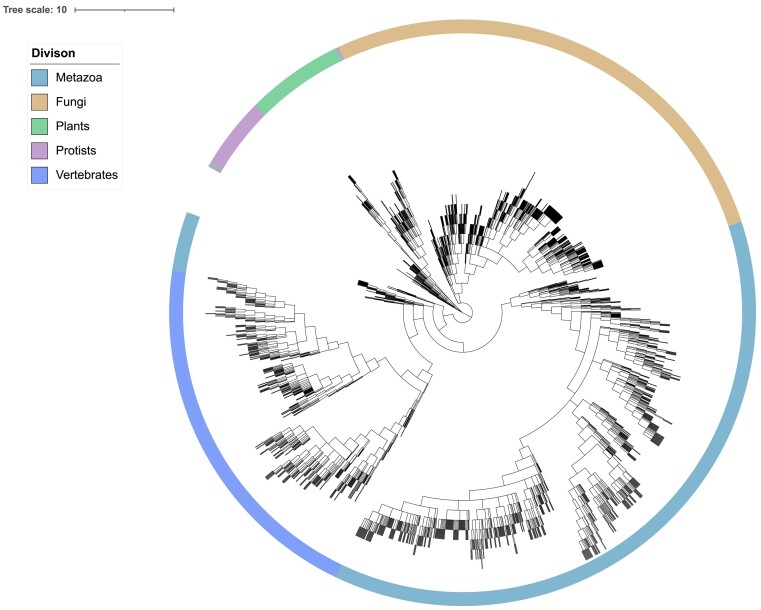
Distribution of eukaryotic species with genomes available across all Ensembl sites. The distribution of eukaryotic species with available genomes from all Ensembl sites is represented based on NCBI taxonomy, highlighting the hierarchical relationships among taxa and their lineages. The tree was estimated through the NCBI interface of ETE3 (43) and annotated using iTOL (44). The coloured circle represents the high-level taxonomic division to which the species in the corresponding region of the tree belong.

Ensembl aims to provide genomes and data of interest to a broad range of stakeholders, covering human, model organisms, species of agricultural and medical relevance, and species from global biodiversity projects. Over the past 12 months, we have added >1000 eukaryotic genome assemblies from >800 species predominantly from invertebrates and plants taking us to >35 000 genomes in total (Figure 1). These additions have been primarily driven by collaborations with biodiversity projects and resources such as VEuPathDB (1), within-species pan-genomes and user requests. One requirement for all genomes that we introduce is that the assembly must be available from the International Nucleotide Sequence Database Collaboration archives (https://www.insdc.org).

We are working towards the development of a single unified Ensembl site, where we plan to integrate all our existing component sites over the coming 2 years. In the interim, we advise users to continue to use the component sites, as these have full Ensembl functionality. However, there are already >2000 assemblies, primarily from insects, animals and plants, which are only available from our new beta site (see the ‘Graphical abstract’ section). We encourage users interested in those species to explore our new site, where we will continue to expand the data and functionality available.

Within the Ensembl Metazoa site, we have added almost 80 new genomes selected to increase the taxonomic coverage of species we host. Notably, 12 new clades were added: a ribbon worm clade (Nemertea), six insect clades (Trichoptera, Liviidae, Conopidae, Ichneumonidae, Braconidae and Acrididae), a non-venomous spider clade (Uloboridae), two decapod crustacean clades (Varunidae and Parastacidae) and two hydrozoan cnidaria clades (Hydractiniidae and Hydridae). We will continue to import and update the key community references we hold, alongside the expansion of taxonomic coverage in coming releases, for example with the addition of species *Cylas formicarius* as a new Coleoptera representative in the family Brentidae (primitive weevils) available in Ensembl release 113. Ensembl Fungi and Protists will also update a number of key gene sets in line with data available from other public fungal resources.

Key genomes for agriculturally relevant plant species have been added into Ensembl Plants, including the cereals: goatgrass (*Aegilops umbellulate*), Timopheev’s wheat (*Triticum timopheevii*) and a chromosome-level assembly of the elite bread-wheat cultivar Paragon (*Triticum aestivum*). In addition, the following legumes have been introduced: wild soybean (*Glycine soja*), peanut (*Arachis hypogaea*), barrel clover (*Medicago truncatula*), faba bean (*Vicia faba*) and grasspea (*Lathyrus sativus*). We have also updated the cassava (*Manihot esculenta*) genome, a staple crop for Africa and Latin America.

As communities move away from having a single reference assembly for their species of interest, Ensembl is increasingly hosting multiple genomes for a given species. For farmed and companion animal genomes, we have annotated and added two horse (*Equus caballus*) breeds (Friesian and Warmblood) and two chicken (*Gallus gallus*) breeds (Cobb and Ross) to the Ensembl main site as part of the EuroFAANG and VGP projects, respectively. We have also annotated additional strains for Atlantic salmon (*Salmo salar*), Atlantic cod (*Gadus morhua*) and three-spined stickleback (*Gasterosteus aculeatus*). We plan to expand the current set of hosted pangenomes by including barley (*Hordeum vulgare*) and oat (*Avena sativa*) in forthcoming releases of Ensembl Plants.

## Annotation updates—human and beyond

### Gene annotations

**Figure 2.**
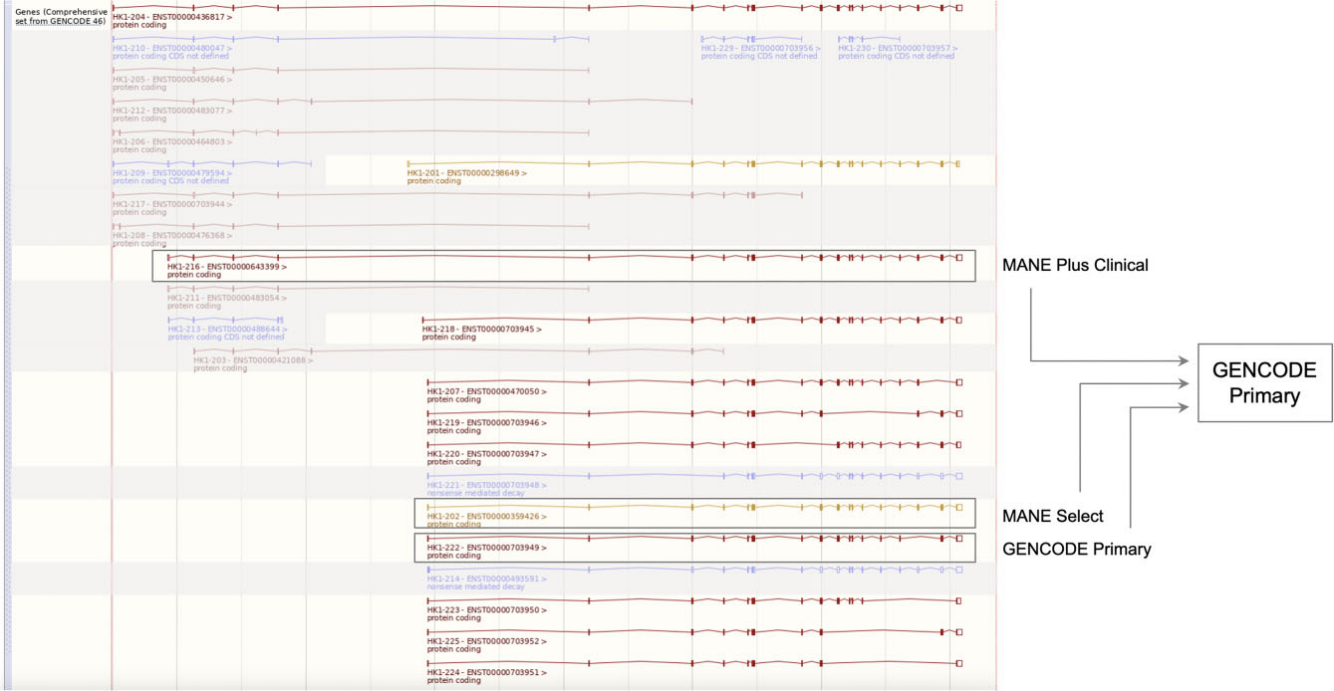
Ensembl location view of the human gene HK1 showing MANE Select, MANE Plus Clinical and GENCODE Primary transcripts. Screenshot of the HK1 gene on human GRCh38 assembly chromosome 10 from Ensembl release 112 (https://www.ensembl.org/Homo_sapiens/Gene/Summary?db=core;g=ENSG00000156515). The GENCODE Primary set will reduce the number of transcripts in the default view to 3 (MANE Select, MANE Plus Clinical and GENCODE Primary) from the complete set of 30 (screenshot truncated) from the GENCODE Comprehensive set. Greyed out transcripts are those not contained within the GENCODE basic set, which has 12 transcripts for this gene.

The GENCODE project aims to identify and classify all gene features in human and mouse, using a combination of manual curation and computational analyses, aided by targeted experimental approaches (2). In Ensembl, GENCODE is the default gene set for human and mouse. The MANE (Matched Annotation from NCBI and EMBL-EBI) project was developed to define a genome-wide set of representative transcripts and corresponding proteins for human protein-coding genes where each transcript is identical in both Ensembl/GENCODE and RefSeq annotation sets and is present in the GRCh38 reference assembly (3). MANE (v1.3 in Ensembl release 112) encompasses >99% of human protein-coding genes and contains 19 288 MANE Select transcripts (one transcript at each protein-coding locus that is representative of biology at that locus), including all genes in the American College of Medical Genetics and Genomics Secondary Findings list v3.2 set (4). This set includes 62 MANE Select transcripts that are annotated on genome patches as they cannot be represented on the primary GRCh38 assembly. The release also included 64 MANE Plus Clinical transcripts, which are additional transcripts added where the MANE Select set is not sufficient to report all pathogenic or likely pathogenic clinical variants (Figure 2). We have also added MANE Select transcripts for a small set of clinically important long non-coding RNAs (lncRNAs). The first set of MANE Select lncRNAs can be found in MANE v1.4 available in Ensembl release 113.

There are currently two Ensembl/GENCODE transcript sets available: one set containing full-length coding transcripts of protein-coding genes, and one transcript per locus for all other gene biotypes including lncRNAs (GENCODE Basic) and one set containing all transcripts (GENCODE Comprehensive). As more full-length isoforms are being annotated based on long-read transcriptome evidence, the Basic set is rapidly expanding. As such, we have introduced a third set, GENCODE Primary, currently restricted to protein-coding genes (Figure 2). This set includes by default the MANE Select Ensembl Canonical and MANE Plus Clinical transcripts. We use biological data (expression and evolutionary constraint) to evaluate the functional potential of features (exons, introns and splice sites) that are not present in the reference (MANE Select) transcript for that locus and possess characteristics suggestive of function. We then run the Ensembl Select pipeline, developed to identify candidate MANE Select transcripts, to find the highest scoring transcript that includes the identified novel functional feature. The pipeline is re-run for each Ensembl/GENCODE release and these additional transcripts have been flagged as GENCODE Primary in the GFF3 files from Ensembl release 112 onwards, with visible flags in the transcript table on gene pages from release 113.

Furthermore, we have annotated the most recent assemblies for 17 inbred mouse strains from the Mouse Genomes Project (5). These annotations were produced using a combination of the Ensembl Mapping pipelines, which were initially developed for annotating human haplotype assemblies in the HPRC project (6), along with alignments of strain-specific transcriptomic data to the genome. Annotations for the mouse strains are currently available via rapid.ensembl.org and will be integrated to Ensembl’s main and beta sites in a forthcoming release. We have also updated the annotation for 14 fish reference species, including three-spined stickleback (*Gasterosteus aculeatus*), Atlantic herring (*Clupea harengus*) and northern pike (*Esox lucius*).

We have significantly expanded our non-vertebrate annotations, adding 60 newly annotated plant genomes to our beta site, following our landmark achievement of annotating the first plant genome using Ensembl pipelines last year. Additionally, we have broadened our focus to include new clades, utilizing the Ensembl automated annotation pipelines to annotate 63 cnidarians, 39 molluscs, 3 bryozoans and 2 echinoderms, among other taxa.

We continue to generate annotations for assemblies from various global diversity initiatives, such as the Darwin Tree of Life project (7), the European Reference Genome Atlas (8) and the Earth Biogenome Project (9), and more recently the Canada Biogenome Project and Aquatic Symbiosis Genomics Project (10) using the Ensembl automated annotation system (https://beta.ensembl.org/help/articles/gene-annotation). Having access to sufficient and diverse RNASeq data is a prerequisite for the annotation process. Ensembl data for these projects are summarized via the Ensembl project pages: projects.ensembl.org.

### Microbial gene annotations

We have released a comprehensive, robust NextFlow (11) pipeline to annotate prokaryotic genomes using numerous tools and databases such as Prokka (12), InterProScan (13) and AMRFinderPlus (14) to predict as much functional information as possible. This can be particularly challenging for some protists due to the sparsity of annotations for certain taxa. A previous version of this pipeline was used to re-annotate all genomes in Ensembl Bacteria and subsequent releases of Ensembl Bacteria will incorporate the updates to the pipeline. The pipeline is containerised and freely available (15).

### Regulatory features

In addition to gene annotation, Ensembl annotates sets of candidate regulatory features for human and mouse, three livestock species [cow (*Bos taurus*), pig (*Sus scrofa*) and chicken (*Gallus gallus*)] and five aquaculture species [Atlantic salmon (*Salmo salar*), turbot (*Scophthalmus maximus*), European seabass (*Dicentrarchus labrax)*, rainbow trout (*Oncorhynchus mykiss)* and common carp (*Cyprinus carpio carpio*)]. Human and mouse annotation sets are based on epigenomic data collected by Roadmap Epigenomics and ENCODE (16); pig and chicken annotations are based on data from GENE-SWitCH (https://www.gene-switch.eu) and a Functional Annotation of Animal Genomes (FAANG) project (17); and cow is also based on data from this FAANG project. Our aquaculture annotation was performed in collaboration with the AQUA-FAANG (18) consortium.

In (19), we described our initial work to migrate to a new regulatory annotation pipeline, which uses merged open chromatin regions as the basis for regulatory features, rather than chromatin segmentation. Over the past year, we have completed this migration by adding enhancers and CTCF features to our new pipeline, and have applied it to five further species, including human and mouse, bringing our total supported species to 10, available in Ensembl release 113. Features are classified as enhancers based on overlap with H3K4me1 or H3K27ac ChIP-seq, or as promoters if they overlap potential transcription start sites from protein-coding and lncRNA transcripts. We also use the presence and absence of open chromatin peaks in different epigenomes (cell types, cell lines or tissues) to predict activity of regulatory features across these epigenomes. This pipeline was employed in Ensembl release 111 to produce updated regulatory feature annotation—including promoters, enhancers and open chromatin regions—for the above mentioned livestock and aquaculture species, with the exception of cow. In release 112, we added minor refinements for turbot and updated our promoter annotations in human to ensure that they align with the 5′ ends of transcripts. The underlying link between gene and promoter is now available for all species as an attribute of the GFF3 annotation files on our FTP site (https://ftp.ensembl.org/pub/current_regulation/). In Ensembl release 113, we have added cow as a supported species, introduced a new motif mapping pipeline, included CTCF features, and updated our human and mouse annotations using the latest pipeline to ensure all our regulatory annotations have been produced using the same approach.

### Molecular interactions

We have continued to incorporate manually curated molecular interaction data from PHI-base (20), PlasticDB (21) and HPIDB (22) onto precisely matched proteins in our bacterial, protist and fungal genomes. After the most recent run of our mapping pipeline, we now have 3973, 3131 and 203 proteins in bacteria, fungi and protists mapped with interactions to a host genome, a host target (in plants, metazoa and vertebrate sites) or a synthetic molecule (e.g. plastic or a drug) identified using its ChEBI (23) identifiers. The major improvements over the past year, aside from an increase by 15% (2111) in the total number of imported interactions, are the inclusion of antimicrobial resistance information from PHI-base (55 interactions) and the revision of our data exchange formats to better align with the latest upgrade in PHI-base to version 5, which encompasses a gene-centric data model and an updated curation tool (24). All of the interaction information can be searched for via our interactions Rest API and visualized on our gene pages.

## Ensembl tools

We have continued to develop and expand the open source tools available via Ensembl, with a focus on the Ensembl Variant Effect Predictor (VEP) (25), expansion of available Repeat Libraries and the Transcript Archive (Tark).

We have enhanced the functionalities of the Ensembl VEP tool for variant annotation and prioritization, responding to community requests and the availability of new techniques and data. Ensembl VEP enables easy access to extensive phenotype and disease associations for both variants and genes. To provide further evidence of possible disease involvement, it can now report information from gene paralogs and orthologs for human variants. The PhenotypeOrthologous module reports any phenotypes associated with mouse and rat genes orthologous to the human gene a variant falls within. The Paralogues module annotates human variants with any phenotype assertions attached to variants at the matching location in a paralogous gene, an approach shown to be valuable in variant interpretation (26).

Ribosome profiling has revealed thousands of novel open reading frames (ORFs) within lncRNAs and the UTRs of protein coding genes, some of which have been shown to be functional and may be of clinical importance (27). We have extended Ensembl VEP to report when variants lie within these ORFs and predict when they will disrupt translation or change the protein sequence. The Ensembl VEP web interface enables annotation against a community-created catalogue of human Ribo-Seq ORFs (28).

We have created multiple Ensembl VEP extensions to integrate new ratings of human variant deleteriousness. We collaborated with DeepMind to integrate AlphaMissense (29) scores of likely variant pathogenicity into Ensembl VEP, releasing it on the day the scores were made public to enable their use as quickly as possible. Additional scores of potential rare missense variant pathogenicity are imported from VARITY (30). Information on possible variant effects on splicing is made available through the integration of SpliceVault (31), which uses Genotype-Tissue Expression data to predict when a variant may cause cryptic donor or acceptor sites or exon skipping. The Ensembl VEP web interface has also been extended to enable annotation of chicken, dog, goat and sheep variants with population allele frequencies and Atlantic salmon, chicken, pig and rainbow trout with predicted variant impact on regulatory elements.

We have continued to build our extensive collection of repeat libraries using RepeatModeler (32) for 6420 genome assemblies, representing 3450 different species. The libraries represent both vertebrate and non-vertebrate species and are available from the EMBL-EBI FTP site (http://ftp.ebi.ac.uk/pub/databases/ensembl/repeats/unfiltered_repeatmodeler/species/).

The Transcript Archive (Tark, https://tark.ensembl.org/) serves as an archive of all human transcript sequences annotated from Ensembl, RefSeq and other sources, including historical gene sets. Tark has been updated to include transcripts from Ensembl release 112, including the MANE Select and MANE Plus Clinical transcripts.

## Integrating Ensembl across the tree of life

The new Ensembl website (https://beta.ensembl.org) has reached its next major milestone, enabling visualization of all genomes previously hosted within our Rapid Release website, bringing the total available genomes to 2737. All new genomes released supporting Ensembl’s growing biodiversity and pangenome efforts are now being made available through the beta site.

**Figure 3.**
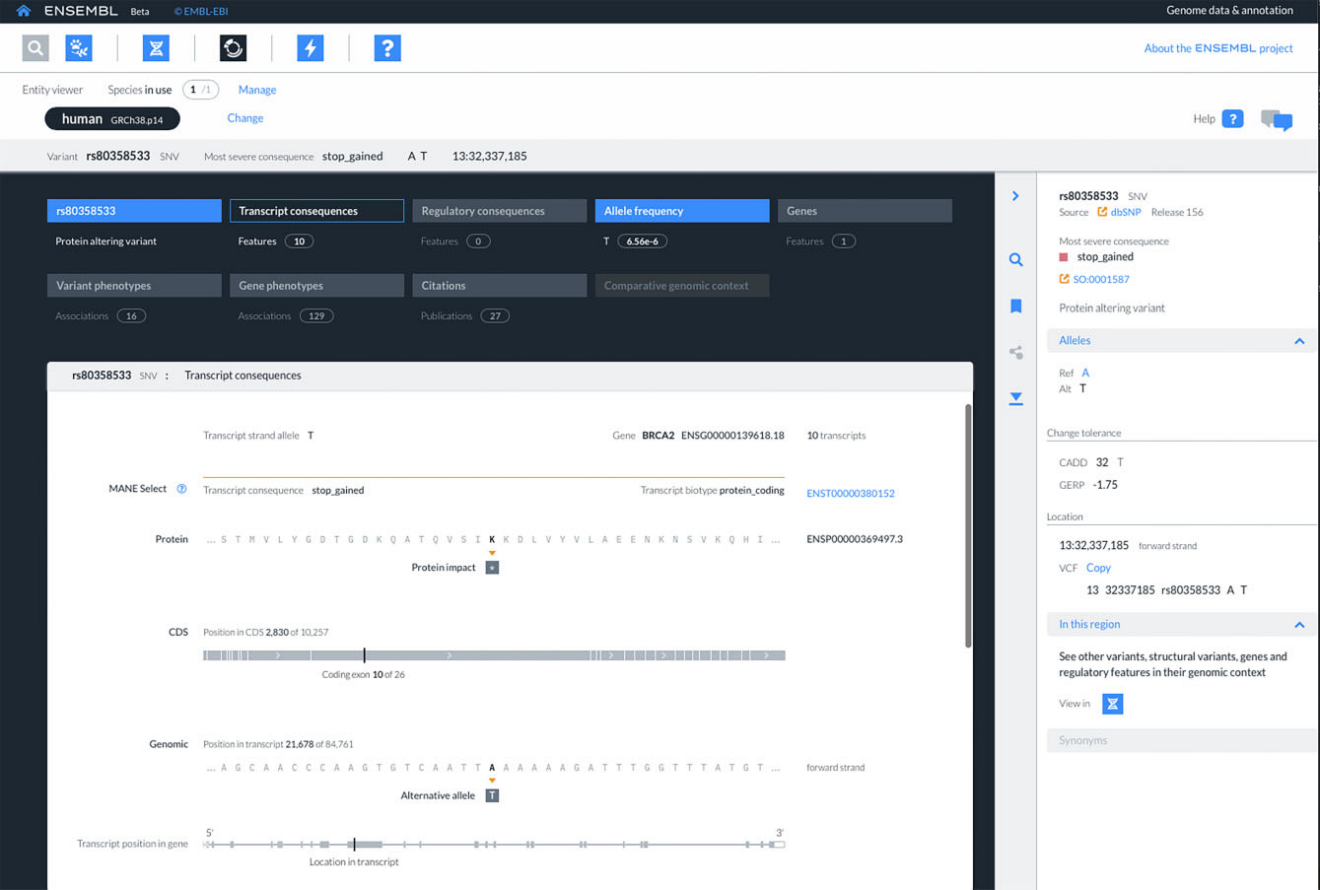
Screenshot of the Variation Entity Viewer overview of transcript consequences tab. The focus variant (rs580358533) is a single nucleotide variant in exon 10 of the BRCA2 gene and the predicted molecular consequence is stop_gained, relative to the MANE select transcript, as shown in the central panel. Allele frequency data are available from the Allele frequency tab, which is the fourth tab from the left on the top. The variant can be viewed at https://beta.ensembl.org/entity-viewer/grch38/variant:13:32337185:rs80358533?allele=0&view=transcript-consequences.

Our beta infrastructure has undergone significant development over the past year: The genome browser interface now includes a new set of tracks including transcription start site predictions, CpG islands, repeat annotation, per-base pair conservation scores and regions of high conservation; homology predictions are now available from the Ensembl entity viewer interface for all genomes; and a new suite of interfaces in our beta infrastructure has been developed to explore single and multiple nucleotide variation events. Ensembl VEP molecular consequences can now be explored with reference to their impact at the genomic, complementary DNA (cDNA), coding sequence (CDS) and protein levels (Figure 3). For human GRCh38, allele frequencies are available from the 1000 Genomes Project Phase 3 (33), gnomAD genomes v3.1.2 (34) and gnomAD exomes r2.1.1 (35) data sets. In addition, GRCh38 variants are annotated with their CADD (36) and GERP (37) scores alongside a VCF representation. Multi-allelic sites are presented in a single summary view with switching between each available allele now a seamless experience. All data are served by a new internal GraphQL service Hypsipyle (https://github.com/Ensembl/ensembl-hypsipyle), which reads data directly from VCF files. Our tools have been expanded to include a new Ensembl VEP interface that enables annotation of user-input variants called against the human pangenome assemblies and biodiversity genomes. This represents the first time Ensembl VEP has been made available as an online tool to enable analysis over these increasingly important data sets.

## Scaling infrastructure to meet demand

The number of genomes being sequenced and published continues to rise as more communities have access to sequencing technologies that enable them to generate high-quality genomes for multiple individuals, even for species with large and/or complex genomes. Ensembl is working to meet the scaling requirements by developing the new infrastructure using an updated technology stack. Our programmatic offerings have been improved with a new set of APIs now available. First, we have provided a public instance of the refget standard (38) from the Global Alliance for Genomics and Health (GA4GH, www.ga4gh.org). Key to refget is the use of checksum identifiers derived from sequence content, meaning retrieval is unambiguous and provides fast access to all genomic, protein, cDNA and CDS sequences hosted in our new infrastructure. The checksum identifiers required for retrieval can be accessed through our GraphQL endpoint with support for MD5 and GA4GH identifiers. As we scaled towards thousands of genomes, we noted scalability challenges in sequence retrieval times, which have been resolved with our new refget implementation (https://github.com/Ensembl/ensembl-refget). This implementation relies on seekable compressed sequence archives and fast lookups into these archives reducing access time to milliseconds from seconds in some cases. Due to the nature of the refget standard, researchers can choose to run a local deployment of our refget server (https://beta.ensembl.org/data/refget/), or any public implementation, negating the need to contact our public services.

Our GraphQL service Thoas (https://beta.ensembl.org/data/graphql) has been expanded to support a new set of metadata endpoints allowing programmatic exploration of Ensembl hosted genomes via a lookup of common identifiers, examination of assembly composition and identification of data sets linked to these genomes. These endpoints allow for programmatic discovery of Ensembl Genome IDs, which are used to identify unique gene set annotations in our APIs. We also provide a resolver service (https://resolver.ensembl.org), which is the recommended mechanism for generating external links into Ensembl’s new infrastructure. The resolver can redirect versioned and unversioned gene stable identifiers with mechanisms for resolving ambiguity where identifiers are shared between assemblies.

## Comparative genomics at scale

Ensembl’s comparative genomics analyses provide our users with mechanisms to explore evolutionary histories of genes, and support the transfer of functional annotations between homologues. Gene trees are generated from the alignments of clustered protein or ncRNA sequences; these are then used to infer homology relationships. In addition, we provide whole genome alignments, which can be pairwise or involve multiple genomes.

Ensembl’s EPO pipeline (39) has been used to generate whole genome alignments, protein trees and ncRNA trees for an expanded pig-breed collection including seven new pig breeds (Bama miniature, Duroc, euw1 european wild boar, NIHS-2020, Ningxiang, Ossabaw miniature and PB115). The wheat cultivar protein trees were also expanded to include four additional cultivars (Kariega, Paragon, Renan and Sy Mattis) and two related *Triticum* genomes (*T. spelta* and *T. timopheevii*). In Ensembl release 111, we completed the first iteration of the move to separated taxonomic protein trees for Metazoa (19), by releasing the new Insecta gene tree, complementing the Metazoa and Protostomia gene trees. We have also added the Drosophilidae gene tree in Ensembl release 112, a genus-level tree containing 36 *Drosophila* genomes and four (Diptera) outgroup species.

Cactus (40) has been used to generate additional multiple whole genome alignments that are displayed on the website, including a 10-member fowl alignment, which also has an updated gene tree collection, and the 40-member Drosophilidae alignment. Cactus alignments (HAL files) are available from our FTP site (https://ftp.ensembl.org).

On the beta site, homology predictions for each species are inferred using a reciprocal best BLAST hit strategy, against a set of species available within the beta infrastructure (https://beta.ensembl.org/help/articles/homology-annotation). The homology data are stored within a MongoDB for performance and delivered to the browser via an internal GraphQL API.

## To learn more about Ensembl

Ensembl offers an extensive training programme including in-person and synchronous virtual courses as well as asynchronous online courses (https://www.ebi.ac.uk/training/on-demand). We continue to deliver virtual courses with open registration for participants across the globe while also collaborating with host organizations to provide training for specific communities, tailored to their needs and interests. Over the past year, 42 in-person and 15 virtual workshops were delivered to over 1100 participants across 14 countries, with a further 10 virtual events open worldwide. Recent examples include a deep dive into the Ensembl workflows for annotating genomes as part of the EURO-FAANG collaboration; sessions within the Livestock Pangenomics summer school at Universita Cattolica, Italy; in-depth tutorials within the Fungal Pathogen Genomics course at Wellcome Connecting Science, UK; and an Ensembl Train the Trainer workshop at Midlands University, Zimbabwe. Course materials are distributed through our training site (https://training.ensembl.org/) and virtual workshops are recorded and hosted on YouTube to share with participants and the wider community. Details of the training we provide and how to host an Ensembl course can be found through our training site and courses with open registration are advertised on the Ensembl blog and social media channels including X, LinkedIn and Facebook.

We continue to offer personalized assistance with specific Ensembl queries on our helpdesk, helpdesk@ensembl.org, and through our developer mailing list (https://lists.ensembl.org/mailman/listinfo/dev).

## Future directions

We will continue to bring new data into Ensembl, particularly related to health, agriculture and biodiversity species. Users may request the addition of specific genomes or data sets by contacting the Ensembl Helpdesk, where the request will be assessed and prioritized according to capacity and scientific demand. We will keep expanding the annotation we provide to new species across the tree of life, and also diversify the variation data sets from the European Variation Archive (41), which we display, showing variants mapped to the assemblies we host. We will continue our active involvement in biodiversity and pangenome efforts to ensure Ensembl resources can support exploration, interpretation and visualization of increasingly complex data sets.

Our next phase of development targets the eventual retirement of our component sites and a full migration towards our new infrastructure. We have prioritized a number of key areas that will help users migrate and continue to use Ensembl effectively and efficiently. First, we are developing our regulation interfaces by bringing in dedicated views similar to those seen for genes and variants by displaying evidence alongside regulation and transcription context. In addition, we plan to migrate our sequence viewer including support for the projection and display of annotation from genomic regions to transcripts and proteins. Such development will also enable integration of AlphaFold (42) into the new site and allow variation projection as offered on our current site. We will also start the migration of our projects pages to the new infrastructure. Finally, we plan to release the beta deployment of our BioMart successor product, offering similar functionality but utilizing an updated technology stack.

The retirement of the Rapid Release website begins a transitional phase for Ensembl where we seek to migrate all sites to our new infrastructure within 2 years alongside additional development needed to port essential functionality from our current infrastructure. While we will continue to maintain archives once a site is retired, no new data will be added and we will only prioritize essential bug fixes. We encourage all Ensembl users to review the functionality on our beta site, provide feedback and contribute to our exciting future.

## Data Availability

All Ensembl integrated data are available without restriction from the main website (www.ensembl.org), the component sites (www.metazoa.ensembl.org, www.plants.ensembl.org, www.fungi.ensembl.org, www.protists.ensembl.org and www.bacteria.ensembl.org), the Rapid Release site (https://rapid.ensembl.org) and Beta Ensembl (beta.ensembl.org), in bulk from the FTP site (https://ftp.ensembl.org) and programmatically via the REST API (https://rest.ensembl.org). Cactus alignments (HAL files) are available from our FTP sites (https://ftp.ensembl.org/pub/rapid-release/data_files/multi/hal_files, https://ftp.ensembl.org/pub/misc/compara/multi/hal_files and https://ftp.ensembl.org/pub/data_files/multi/hal_files). Ensembl code is available from GitHub (https://github.com/Ensembl) under an open source Apache 2.0 licence. News about our releases and services can be found on our blog (https://www.ensembl.info), our announce mailing list (https://lists.ensembl.org/mailman/listinfo/announce), X (@ensembl; https://x.com/ensembl), LinkedIn (https://www.linkedin.com/company/ensemblgenomebrowser) and Facebook (https://facebook.com/Ensembl.org).
